# 

**Published:** 1991-12

**Authors:** 


W1 WE403

V.106 NO.4 1991

C.01--------SEQ: SR0068788

TI: WEST OF ENGLAND MEDICAL

JOURNAL

### WEST OF ENGLAND MEDICAL JOURNAL

Volume 106(iv) December 1991

### HISTORY OF MEDICINE NUMBER

Who wrote the Hippocratic Oath?
The Unwrapping of the Bristol Mummy
The Care of the Newborn in Antiquity
Epilepsy, Brain and Mind
Captain Thomas Dover
Childbirth, Lessons from the Past
The Leonardo Anatomical drawings
Caleb Hillier Parry
The American Prisoners in Dartmoor 1813-1815
Pathos and Pathology in the life of Lord Byron
Serendipity and Logic in Medical Research
Paul Ehrlich and Almroth Wright
Archibald Menzies, Surgeon Botanist
History of the Medical Services in
Weston-super-Mare
Lunacy Bristol Fashion
The Burdens and Bristol
From our Correspondents
Letter to the Editor
Obituary
Book Reviews
South West Orthopaedic Club

